# Equine Methicillin-Resistant Sequence Type 398 *Staphylococcus aureus* (MRSA) Harbor Mobile Genetic Elements Promoting Host Adaptation

**DOI:** 10.3389/fmicb.2018.02516

**Published:** 2018-10-24

**Authors:** Birgit Walther, Katja-Sophia Klein, Ann-Kristin Barton, Torsten Semmler, Charlotte Huber, Roswitha Merle, Karsten Tedin, Franziska Mitrach, Antina Lübke-Becker, Heidrun Gehlen

**Affiliations:** ^1^Centre for Infection Medicine, Institute of Microbiology and Epizootics, Freie Universität Berlin, Berlin, Germany; ^2^Advanced Light and Electron Microscopy (ZBS4), Robert Koch Institute, Berlin, Germany; ^3^Equine Clinic, Surgery and Radiology, Freie Universität Berlin, Berlin, Germany; ^4^Microbial Genomics (NG1), Robert Koch Institute, Berlin, Germany; ^5^Institute for Veterinary Epidemiology and Biostatistics, Freie Universität Berlin, Berlin, Germany; ^6^Faculty of Environment and Natural Sciences, Institute of Biotechnology, Brandenburg University of Technology Cottbus-Senftenberg, Cottbus, Germany

**Keywords:** MRSA, multi-drug resistance, horses, one health, host range, ST398

## Abstract

Continuing introduction of multi-drug resistant, zoonotic pathogens such as methicillin-resistant *Staphylococcus aureus* (MRSA) in horse clinics challenges the biosafety of employees and animal patients. This study was aimed to determine the occurrence of mobile genetic elements facilitating survival in the early stages of invasive infection in different host species, including humans and horses, in MRSA carried by equine patients admitted to a large horse clinic. A total of 341 equine patients were investigated for carriage of MRSA by hygiene screening directly at hospital admission. MRSA were further investigated by antimicrobial susceptibility testing, whole-genome sequencing and genomic composition, including virulence factors involved in immune evasion and host adaption. From a total of 340 validated specimens from equine nostrils, 3.5% yielded positive results for MRSA. All MRSA were found to be closely related belonging to sequence type (ST) 398_t011 with up to four additional antimicrobial resistances. All MRSA harbored a specific Staphylococcal Pathogenicity Island (SaPIbov5) involved in facilitating survival in ruminant and equine plasma. Moreover, a β-hemolysin (*hlb*) converting ΦSa3 phage encoding the human-specific Immune Evasion Cluster (IEC) was present in 72% of the isolates. An equid-specific leukotoxin encoded by a further temperate phage (Saeq1) was only rarely detected (22%). Despite the absence of β-hemolysin production for all IEC-positive ST398, a prominent hemolysis zone was demonstrable on sheep blood agar. Thus, IEC might remain undetected among the ST398 lineage, since the presence of IEC is commonly associated with reduction of hemolysis in *S. aureus* belonging to other genetic backgrounds. Here we describe MRSA-ST398 harboring different mobile genetic elements encoding variants of immune evasion factors and toxins previously shown to contribute to *S. aureus* invasive diseases in specific host species or ecologic niches. We suggest these combinations contribute to the adaptation of MRSA belonging to ST398 with respect to epidemic spread across different habitats and hosts, and may therefore confer a host “generalist” phenotype.

## Introduction

Epidemic and zoonotic methicillin-resistant *Staphylococcus aureus* (MRSA) lineages are characterized by the capacity to colonize and infect a broad host spectrum and circulate within distinct ecological niches (Walther et al., 2009, 2017; Vincze et al., 2014). Recent studies suggested that the dominant European livestock-associated *S. aureus* lineage belonging to sequence type (ST) 398 could increase its threat to public health by continued acquisition of virulence and antibiotic resistance genes (Diene et al., 2017).

Virulence-associated factors promoting colonization and infection and involved in counteracting the host's innate immune defense are often located on mobile genetic elements (MGE) such as pathogenicity islands (SaPI), temperate phages, and plasmids (Everitt et al., 2014; Diene et al., 2017). Examples include phages such as Saeq1 harboring a leukocidin (LukPQ) with a potent and specific killing activity toward equine neutrophils (Koop et al., 2017), and SaPIbov-encoded variants of von Willebrand factor-binding protein (vWbp) (Viana et al., 2010). Together with coagulase (Coa), chromosomally-encoded vWbp form a macromolecular complex with prothrombin, fibrinogen, factor XIII, and fibronectin in human plasma (McCarthy and Lindsay, 2013). The resulting staphylococcal clots are fibrin barriers against host phagocytes, providing a niche for local population development (Thomer et al., 2013). It has been suggested that genes encoding vWbp (vwb) might be a factor involved in animal adaptation, since the vWbp encoded on SaPIbov4 showed specific activity toward ruminant and equine plasma (Viana et al., 2010).

However, additional host defenses against invading bacteria are the recognition, opsonisation, and elimination functions of the complement system (Foster, 2005). Distinct phylogenetic *S. aureus* lineages evade host immune responses differently, with adaption to new host environments often accompanied by acquisition of MGEs and the immune evasion protein complexes that they encode (McCarthy and Lindsay, 2013). The immune evasion cluster (IEC) is commonly associated with beta-hemolysin (*hlb*) converting phages (van Wamel et al., 2006). As a result, IEC-positive *S. aureus* with a disrupted *hlb* often lack a prominent hemolysis zone on sheep blood agar plates (Katayama et al., 2013).

IEC are generally regarded as human-specific, since immune-modulating factors such as the complement-protein cleaving Staphylokinase (*sak*) and the Staphylococcal complement inhibitor (*scn*) inhibit complement factor C3 activation in the human host (McCarthy and Lindsay, 2013). Additional toxins, adhesins, coagulation- and immunomodulatory factors are frequently found to be encoded by genes within SaPIs (Malachowa and DeLeo, 2010).

Here we report on MRSA-carriage rates recorded for horses at hospital admission, the genetic relationship of these isolates and the presence of genes located on MGEs encoding factors involved in establishment of infection in distinct host species.

## Materials and methods

### Inclusion criteria for equine specimens

All specimens were obtained from horses by hygiene screening, directly at hospital admission at the Equine Clinic, Surgery and Radiology, Department of Veterinary Medicine, Freie Universität Berlin, as described earlier (Walther et al., 2018), following the regulations and approval of the Landesamt für Gesundheit und Soziales (LAGESO), Berlin (14.07.2014). Inclusion criteria for all equine samples were defined as: sterile cotton swabs with Amies transport medium (Mast Diagnostica, Reinfeld) of both anterior nostrils taken on arrival without delay and fecal samples taken within 120 min after admission (Walther et al., 2018).

### Statistical analysis

Data were analyzed using IBM SPSS version 24. Differences in the occurrence of positive nostril swabs between the “colic” group and the “open wound” group were investigated using the chi-square-test. *P*-values below 0.05 were considered statistically significant.

### Microbiological approach and antimicrobial susceptibility testing

Fecal specimens, nostril- and wound swabs were initially cultured on Columbia agar with 5% sheep blood (bioMérieux, Germany), and chromID® MRSA (bioMérieux, Germany) agar plates. Identification of *S. aureus* was determined by MALDI-TOF MS (Bruker, Germany). Wound swabs were taken immediately upon admission from the “open wound” patients within the reception area. Further information on the complete sampling procedure and local setting has been published elsewhere (Walther et al., 2018). Antimicrobial susceptibility testing (AST) using the VITEK®2 system (BioMérieux, Germany) was performed according to the standards given by the CLSI VET01-A4 and M100-S21 (Clinical Laboratory Standards Institute, 2011, 2013).

#### CAMP test

The CAMP test (originally described by Christie et al., 1944) is commonly used to identify Group B streptococci, which secrete a protein called CAMP factor known to interact with the β-hemolysin of *S. aureus* (Spellerberg and Brandt, 2015). All isolates were tested for the CAMP phenomenon using a diffusion test on Columbia agar with 5% sheep blood (bioMérieux, Germany), with the quality control strain *Streptococcus agalactiae* (ATCC12386), and the beta-toxin-producing *S. aureus* strain ATCC25923 as a positive control. Note that β-hemolysin enhances lysis by δ-hemolysin, but inhibits lysis by α-hemolysin (Traber et al., 2008). After 18 h at 37°C incubation followed by 4 h at 4°C, isolates were inspected for CAMP hemolysis.

### Whole genome sequencing of equine MRSA

MRSA isolates were whole-genome sequenced (WGS) using Illumina MiSeq 300 bp paired-end sequencing with an obtained coverage > 90X. Adapter-trimmed reads were used for *de novo* assembly into contiguous sequences (contigs) and subsequently into scaffolds using SPAdes v3.11. All draft genomes were annotated using Prokka (Seemann, 2014). WGS data were used for genotypic characterization including the determination of the sequence type (ST) with online tools (https://cge.cbs.dtu.dk/services) including MLSTFinder, *spa*Typer 1.0 and ResFinder 2.1 (threshold: 95% ID, 80% minimum length) for transferable resistance gene detection (Larsen et al., 2012; Zankari et al., 2012; Bartels et al., 2014). Genomic integration sites of mobile genetic elements and amino acid exchanges in resistance-associated genes were investigated using Geneious 10.0.5 (Biomatters Ltd., Australia).

#### Maximum likelihood tree based on determination of the maximum common genome (MCG)

In order to compare the genomes at high resolution, we used the maximum common genome (MCG) that is defined by those orthologous genes present in all considered genomes (von Mentzer et al., 2014). The coding sequences were clustered based on the parameters of sequence similarity (min. 70%) and coverage (min. 90%). The MCG was defined as those genes that were present in each genome and fulfilled the threshold parameters, yielding 2,346 genes. Allelic variants of these genes were subsequently extracted from all genomes by an in-house developed blast-based pipeline, then aligned individually for each gene and concatenated, resulting in an alignment of 1.981 Mbp for these strains. The alignment was used to generate a maximum likelihood phylogenetic tree using RAxML 8.1 (Stamatakis, 2014), including livestock-associated methicillin-resistant *S. aureus* strain 08S00974 (accession number CP020019) isolated from a pig on a fattening pig farm in Germany (Makarova et al., 2017) as an outgroup.

The resulting alignment was screened for pairwise single nucleotide polymorphism (SNP) differences and based on these values a SNP-distance matrix was created.

## Results

We prospectively investigated equine patients admitted to the equine clinic of the Freie Universität Berlin, Department of Veterinary Medicine, which showed clinical signs associated with either “colic” or “open wounds,” from April through October in 2014, and again in 2015. All horses were screened for colonization/infection with multidrug resistant and zoonotic bacteria to develop hygiene improvement strategies (Walther et al., 2018).

### Carriage rates and antibiotic resistance among MRSA from equine patients at hospital admission

MRSA carriage was investigated in 341 equine patients (Table 1). Information on the inclusion/exclusion criteria for horses has been previously described (Walther et al., 2018). One nostril swab and 23 fecal samples were excluded due either to time delays in sampling post-admission or because they were not available.

**Table 1:** Results from MRSA screening of equine patients at hospital admission.

**Specimens (admission)**	**Screening period I & II**						**Screening period I**						**Screening period II**					
	***n*** = **341 horses**						**Apr-Oct 2014 (*****n*** = **174 horses)**						**Apr-Oct 2015 (*****n*** = **167 horses)**					
	**Total (I**+**II)**		**Colic^a^**		**Open wound^b^**		**Total (I)**		**Colic^a^**		**Open wound^b^**		**Total (II)**		**Colic^a^**		**Open wound^b^**	
	***n***	**%**	***n***	**%**	***n***	**%**	***n***	**%**	***n***	**%**	***n***	**%**	***n***	**%**	***n***	**%**	***n***	**%**
Nostril swabs (valid)	340	100	232	100	108	100	173	100	113	100	60	100	167	100	119	100	48	100
MRSA	12	3.5	10	4.3	2	1.9	5	2.9	3	2.7	2	3.3	7	4.2	7	5.9	0	0
Feces (valid)	318	100	220	100	98	100	166	100	109	100	57	100	152	100	111	100	41	100
MRSA	2	0.6	1	0.5	1	1.0	2	1.2	1	0.9	1	1.8	0	0	0	0	0	0
Wound swabs					108	100					60	100					48	100
MRSA					4	3.7					3	5.0					1	2.1

n, number; MRSA, methicillin-resistant S. aureus.

a horses admitted to the horse clinic with clinical signs associated with the colic complex.

b *horses admitted to the horse clinic with an open wound*.

Out of a total of 340 validated nostril swabs, 3.5% were found positive for MRSA, with a slightly higher rate (4.3%) among the “colic” group in comparison with the “open wound” group (1.9%), but were not statistically significant (*p* = 0.253, chi-square-test). Four wound swabs taken from horses of the latter group (*n* = 108) were MRSA-positive (3.7%). Moreover, 318 validated fecal samples were obtained, with an isolation rate of 0.6% MRSA.

Antibiotic susceptibility testing results revealed additional resistances to aminoglycosides (gentamicin, kanamycin) and tetracycline for all 18 MRSA tested, with 66.7% of the isolates also showing resistance toward fluoroquinolones (enrofloxacin, marbofloxacin) and 61% to trimethoprim-sulfonamide (Table 2). Whole genome sequence data yielded corresponding resistance genes including *bla*Z and *mec*A (penicillin/methicillin resistance) *tet*(M) for tetracycline resistance, *aac*A-*aph*D (aminoglycoside resistance), and the trimethoprim resistance gene *dfr*K located on transposon Tn559 (Kadlec and Schwarz, 2010). Moreover, all genomes harbored *str*A (streptomycin resistance). Phenotypic resistance toward fluoroquinolones was associated with amino acid exchanges in the gyrase and topoisomerase IV at the following positions: Ser84Leu in GyrA, Glu182Asp in GyrB, Ser80Phe, Val590Ile and Val656Ile in GrlA and Ile72Thr, Glu422Asp and Glu596Asp in GrlB, matching a profile described only recently for MRSA-ST398-t011 isolated from specimen of equine ocular surfaces (Soimala et al., 2018).

**Table 2:** Resistance profiles and virulence characteristics of 18 MRSA-ST398 isolated from horses at hospital admission.

**No.^a^**	**IMT _ID^b^**	**Date**	**IEC^c^**	***luk*P/Q^d^**	**CAMP^e^**	**Resistance phenotype^f^**
1	33368	May 2014	pos.	neg.	neg.	GEN, KAN, TET, SXT
2	33391	May 2014	pos.	neg.	neg.	GEN, KAN, TET, ENR, MAR
3	33826	Jul 2014	pos.	neg.	neg.	GEN, KAN, TET, ENR, MAR, SXT
4	33828	Jul 2014	pos.	neg.	neg.	GEN, KAN, TET, ENR, MAR
5	33861	Jul 2014	pos.	neg.	neg.	GEN, KAN, TET, ENR, MAR
6	33862	Jul 2014	pos.	neg.	neg.	GEN, KAN, TET, ENR, MAR
7	33997	Aug 2014	pos.	neg.	neg.	GEN, KAN, TET, ENR, MAR, SXT
8	34080	Aug 2014	pos.	neg.	neg.	GEN, KAN, TET, ENR, MAR, SXT
9	34209	Sep 2014	neg.	neg.	pos.	GEN, KAN, TET, SXT
10	34426	Sep 2014	pos.	neg.	neg.	GEN, KAN, TET, ENR, MAR, SXT
11	36995	Jun 2015	pos.	neg.	neg.	GEN, KAN, TET, ENR, MAR
12	37082	Jul 2015	neg.	pos.	pos.	GEN, KAN, TET, SXT
13	37264	Aug 2015	pos.	neg.	neg.	GEN, KAN, TET, ENR, MAR
14	37277	Aug 2015	pos.	neg.	neg.	GEN, KAN, TET, ENR, MAR
15	37325	Aug 2015	neg.	pos.	pos.	GEN, KAN, TET, SXT
16	37340	Aug 2015	neg.	pos.	pos.	GEN, KAN, TET, SXT
17	37426	Sep 2015	pos.	neg.	neg.	GEN, KAN, TET, ENR, MAR, SXT
18	37510	Sep 2015	neg.	pos.	pos.	GEN, KAN, TET, SXT

All MRSA-ST398-t011 of equine origin harbor SaPIbov5 (1–18).

a Number used to identify isolates in Figures 3, 4.

b Strain collection number at Institute of Microbiology and Epizootics (IMT), Freie Universität Berlin.

c Presence of a beta-hemolysin converting ΦSa3 phage (40 kb) harboring a variant of the Immune Evasion Cluster (Figure 2).

d Presence of 45 kb prophage (ΦSaeq1) known to harbor a leukocidin (LukPQ) with potent and specific killing activity toward equine neutrophils.

e CAMP test result: enlarged area of hemolysis formed by β-hemolysin production from Staphylococcus aureus (Figure 4).

f *Results from antimicrobial susceptibility testing (technical appendix) by VITEK®2 system (BioMérieux, Nürtingen, Germany) for non-beta-lactam antibiotics. GEN, gentamicin; KAN, Kanamycin; ENR, enrofloxacin; MAR, marbofloxacin; TET, tetracycline; SXT, trimethoprim-sulfamethoxazole*.

### Phylogenetic relationship of MRSA ST-398 isolated from equids

All MRSA were submitted for whole-genome sequencing (WGS) using Illumina MiSeq 300 bp paired-end sequencing, and screened for virulence- and resistance factors. The accession numbers of the current strains are listed in Supplemental Table 1. All MRSA belonged to the so-called livestock-associated lineage predominating in Europe (ST398, *spa* type t011), and harbored a complete SCC*mec*IV element and SaPIbov5 (Viana et al., 2010).

**Figure 1:**
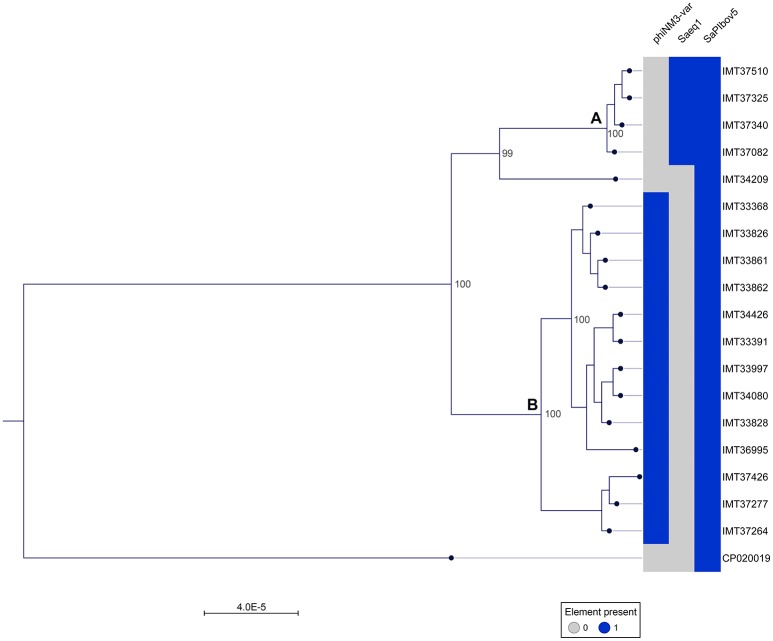
Close relationship of 18 equine MRSA-ST398-t011 isolated from horses at hospital admission. Maximum Likelihood Phylogeny: Phylogenetic tree generated with RAxML, displayed with CLC Genomics Workbench on the basis of 2,346 orthologous genes from 19 ST398 *S. aureus* WGS, including livestock-associated methicillin-resistant *Staphylococcus aureus* (ST398) strain 08S00974 (CP020019) as an outgroup. Presence of mobile genetic elements harboring host adaptation factors is marked blue. phiNM3-var., ΦSa3 phage (40 kb) harboring an immune evasion cluster (IEC); Saeq1, 45 kb prophage Φ Saeq1; SaPIbov5, *Staphylococcus* pathogenicity island SaPIbov5. Two subgroups could be distinguished, cluster **(A)** and **(B)**.

A phylogenetic tree was generated from the MCG comprising 2,346 orthologues genes including WGS data (CP020019) of a livestock-associated MRSA-ST398 strain (08S00974) as an outgroup. Overall, all 18 equine MRSA strains appeared closely related. However, separation of two distinct clusters was possible based on the core genome phylogeny (Figure 1). Pairwise distance ranges among the MCG were calculated for all equine MRSA isolates (Supplemental Table 2), yielding 0-1 SNPs for the most related strains (cluster A, Figure 1), 0-43 SNPs for the MRSA clustering together within B, and 60-92 SNPs for a singleton, representing the most distantly related strain (IMT34209) in this study. A range of 187-201 SNPs was calculated based on the WGS data of the outgroup strain (08S00974), which is also positive for SAPIbov5 and shares the ST398 background.

**Figure 2:**
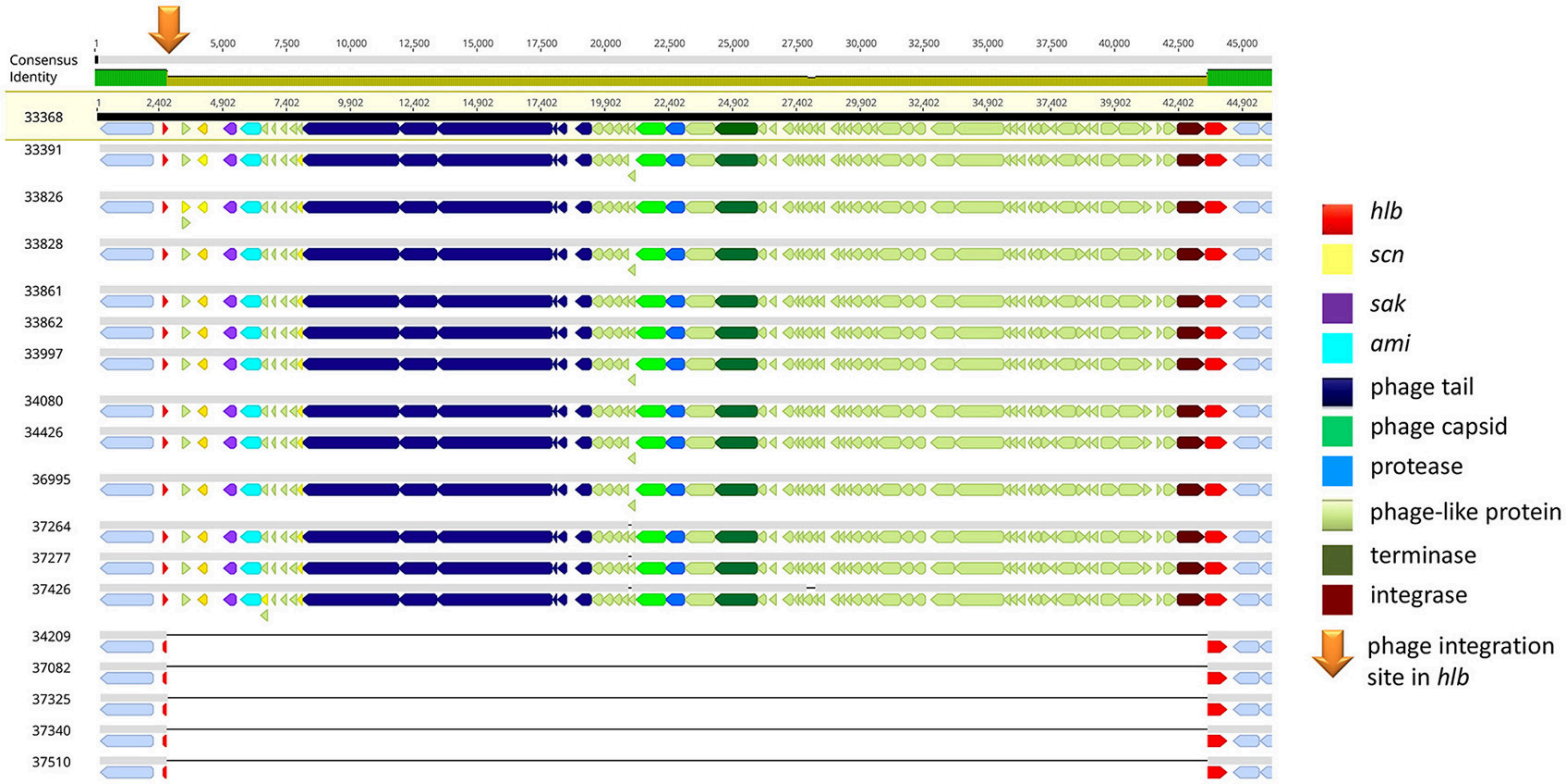
IEC-positive phiNM3-like phage integrated in *hlb* gene of equine MRSA-ST398. Gene map of the *hlb-*encoding phospholipase C (ß-hemolysin) region in equine MRSA-ST398. The colored bar at the top indicates the mean pairwise nucleotide sequence identity in the column: bright green = 100% identity; green-brown = <100% but >30% base pair identity. Prophage genes are colored based on putative or known function. Genomic attachment sites were shown in gray. The integration site (5′-GTATCCGAATTGG-3′) of 40,699 bp or 40,703 bp phiNM3-like phages (coverage: 96%, similarity 98%; DQ530361.1) harboring genes of the Immune Evasion Cluster (*scn, sak*) within the first 13 genomes is indicated by an orange arrow, resulting in a disrupted *hlb* gene (red) of 301 bp (left) and 825 bp (right). So far, ΦSa3 integration in *hlb* of ST-398 was rarely reported (Kraushaar et al., 2017).

Of note, four MRSA identified in 2015 were positive for a recently described temperate phage, Saeq1 (Table 2). In 13 equine MRSA-ST398, a ΦSa3 phage of 40 kb length closely related to phiNM3 (DQ530361.1; coverage: 96%, similarity 98%) was found to have a disrupted β-hemolysin gene, *hlb* (Figure 2). A single strain (IMT34209) lacks both MGEs. The presence/absence of these MGEs matches with the core genome phylogeny (Figure 1).

All strains harbored two allelic variants of *vwb*, a chromosomally-encoded gene of 1,515 base pair length sharing 100% identity with *vwb* of HM240418.1 and a further variant located on SAPIbov5, which is 100% similar to the *vwb* gene reported for SaPIbov4 (HM211303.1) (Diene et al., 2017).

### Hemolysis on sheep blood agar plates

**Figure 3:**
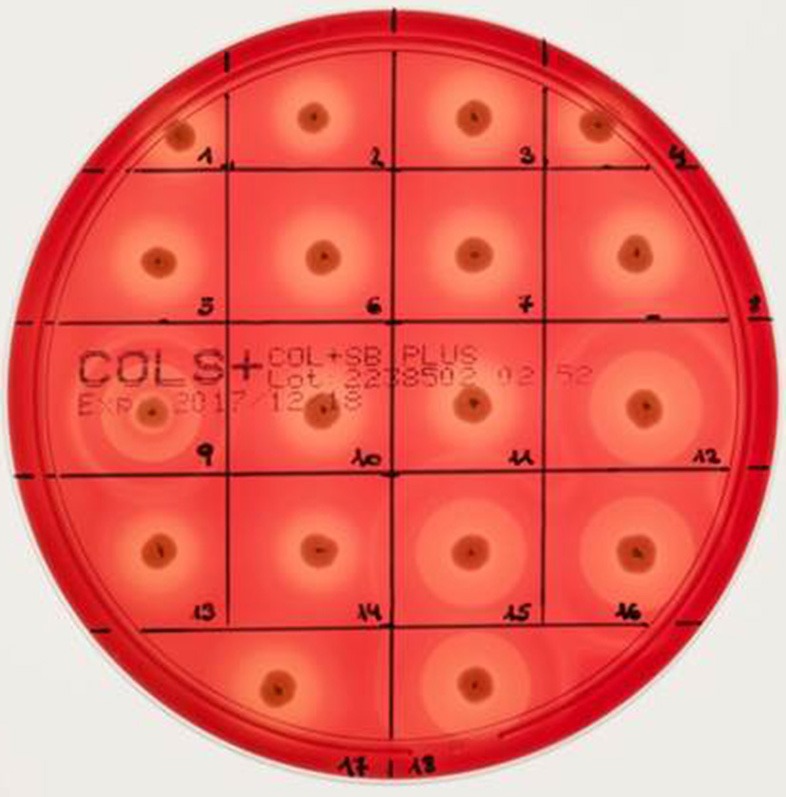
Hemolysis of equine MRSA-ST398 on sheep blood agar plate. Hemolysis zones of closely related MRSAST398-t011 isolated from horses on sheep blood agar plates after 18 h at 37°C incubation followed by 4 h at 4°C. The β-hemolysin activity induced “double zone” hemolysis (halo) was noticed only for isolate 9, 12, 15, 16, and 18. Isolates 1–8, 10, 11, 13, 14, and 17 harbor a β-hemolysin disrupting phage carrying IEC (numbers as indicated in Table 2).

**Figure 4:**
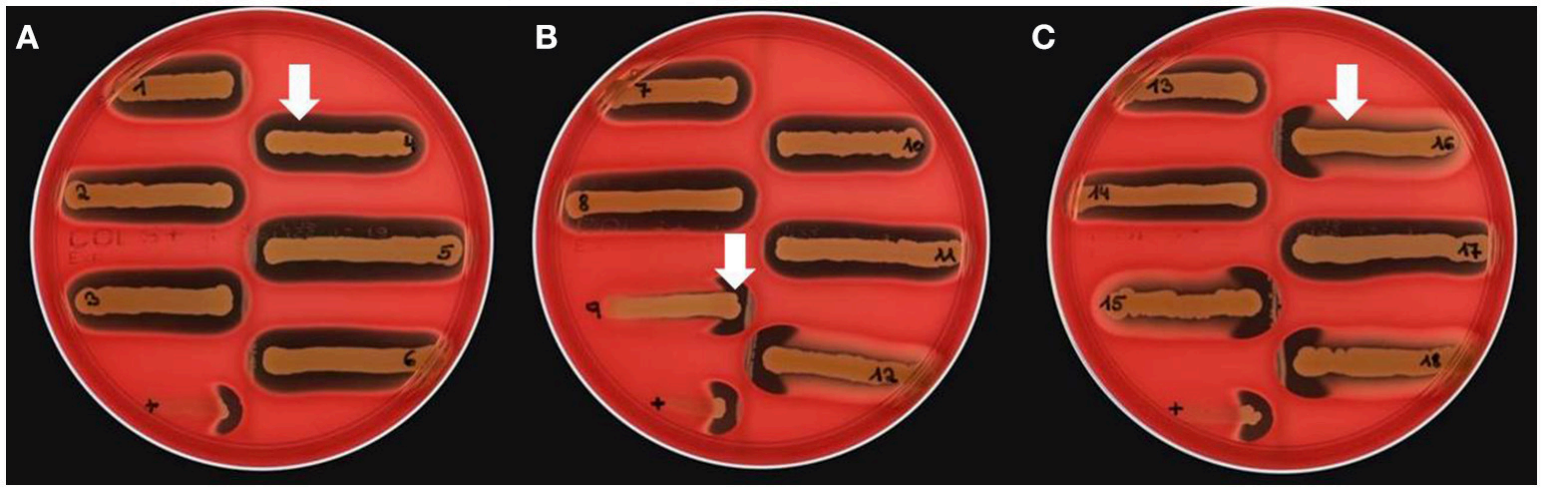
Lack of β-hemolysin production in MRSA-ST398 harboring IEC. **(A–C)** CAMP test results for *Streptococcus agalactiae* (grown as central diameter on each plate) together with 18 MRSA-ST398-t011 isolates of equine origin. grown as central diameter on each plate) together with 18 MRSA-ST398-t011 isolates of equine origin. **(A)** White arrow: large hemolytic zone irrespectively of CAMP factor presence in 1–8, 10, 11, 13, 14, and 17. These isolates harbor a β-hemolysin disrupting phage carrying IEC (numbers as indicated in Table 2). **(B)** White arrow: enlarged hemolysis zone induced by synergistic activity of CAMP factor- and β-hemolysin production in 9, 12, 15, 16, and 18. **(C)** White arrow: β-hemolysin production inhibits lysis by α-hemolysin (Traber et al., 2008), resulting in weaker total hemolysis zones for the IEC-negative isolates 9, 12, 15, 16, and 18. +, positive control S. *aureus* ATCC 25923.

Different bi-component leucocidins including γ-hemolysin, the pore forming toxins α-hemolysin (*hla*), β-hemolysin and phenol soluble modulins, are among the factors which determine the hemolytic appearance of *S. aureus* on sheep blood agar (SBA) plates. Despite an explicit hemolysis zone on SBA plates for all MRSA-ST398 isolates reported here (Figure 3), the lack of β-hemolysin production (phospholipase C, a sphingomyelinase) was shown by employing a CAMP cross-streaking test using the CAMP factor produced by *Streptococcus agalactiae* (ATCC12386) as an indicator for β-hemolysin production. As shown in (Figure 4), the absence of β-hemolysin production was verified by the lack of a CAMP phenomenon in all MRSA-ST398 possessing the ΦSa3 phage, although a relatively large hemolysis zone is apparent. In addition, β-hemolysin production inhibits lysis by α-hemolysin (Traber et al., 2008), resulting in weaker total hemolysis zones for the IEC-negative isolates 9, 12, 15, 16, and 18 (Figures 4B,C).

## Discussion

Here we provide evidence not only for the continuing entrance of MRSA into veterinary clinics through colonized and/or infected horses, but also additional molecular evidence for contemporary carriage of MGEs within the so-called “live-stock” MRSA-ST398 lineage— MGEs encoding virulence factors previously found to be involved in adaption to different host species.

### Carriage of multi-drug resistant MRSA-ST398-t011 in horses at hospital admission

Similar to the large geographic variations for MRSA detection rates reported for human patients at hospital admission, MRSA (nostril-)screening results for horses at hospital admission have shown large variations in former studies, generally ranging from 2.9 to 10.9% (Tokateloff et al., 2009; Weese and van Duijkeren, 2010). In this study, the average nostril-swab detection rate was 3.5%. However, the colic group yielded more MRSA-positive isolates than the open wound group (4.3 vs. 1.5%), but the difference was not statistically significant. Since horses often suffer from recurrent colic, prior episodes of intensive veterinary care or hospital stays might have an influence on the detection rate for this group. Unfortunately, long-term retrospective data was not available for the horses presented here and should be included in further studies. While *S. aureus* fecal carriage has been identified as a potential source for nosocomial transmission and a risk factor for disease development in human medicine (Claassen-Weitz et al., 2016), the detection rate of 0.6% for horses at hospital admission seems to be of less importance with respect to equine hospital hygiene. In contrast, in human medicine, intestinal carriage seems of much greater importance and has been assumed to be generally underestimated (Acton et al., 2009).

All MRSA isolates reported here revealed a remarkable multi-drug resistance, comprising up to five different classes of antimicrobials (Table 2). A further study including horses with acute or chronic ocular disorders presented at the horse clinic of the Freie Universität Berlin in 2015/2016 revealed the occurrence of closely related, multi-drug resistant MRSA-ST398-t011 in samples from ocular surfaces as well (Soimala et al., 2018).

While MRSA-ST398 were infrequently reported as cause of human infections (van Duijkeren et al., 2011; Kinnevey et al., 2014; Larsen et al., 2015), hospitals located in pig population-dense regions appear to be a hot-spot for this genotype (Köck et al., 2009, 2013; van Alen et al., 2017).

### Phylogenetic relationship of MRSA-ST398 isolated from hospitalized horses

Although the occurrence of different genotypes has previously reported in prior studies (Cuny et al., 2006, 2008; Walther et al., 2009), more recent studies, including this study, revealed an absolute predominance of MRSA-ST398 in samples obtained from horses (Vincze et al., 2014).

In recent years, several reports indicated not only the occurrence of phages harboring the IEC in MRSA-ST398 of equine origin (Cuny et al., 2015; Islam et al., 2017), but also a possible beneficial impact of IEC-carriage in MRSA-ST398 on bacterial survival in the presence of human and equine polymorphic neutrophils (Jung et al., 2017).

Here, we report on WGS data from MRSA isolates of 18 horses sampled directly at hospital admission, before entering the hospital stables or other rooms. The core genome together with the SNP ranges calculated based on the 2,346 bp MCG of these 18 MRSA-ST398-t011 revealed a close phylogenetic relationship, mirrored by range of 0-92 SNPs considering pairwise distance ranges for all 18 equine MRSA (Supplemental Table 2). However, two subgroups (cluster A and B in Figure 1) and a singleton (IMT34209) could be distinguished, yielding SNP ranges from 0 to 1 within cluster A and 0–43 in cluster B. A former study on MRSA from veterinary clinics in the UK revealed that isolates from a particular clinic often clustered together on the phylogenetic tree, including different clades of closely related isolates from a broad range of infections (Harrison et al., 2014). Interestingly, the presence or absence of either Saeq1 or an IEC-carrying phiNM3-like phage, which were not part of the core genome analysis, matches strictly with the phylogenetic tree (Figure 1).

A recent study investigating WGS data of LA-MRSA belonging to clonal complex 398 revealed some evidence of phylogeographic patterns with a majority of European isolates clustering together and forming a unique lineage compared with the non-European isolates (Sharma et al., 2016). The largest European sub-lineage, denominated as EU t011, was further separated into two branches: One group harboring SCC*mec*IV and the other SCC*mec*V (Sharma et al., 2016). Interestingly, transferable resistance genes detect in this study were similar to the profiles reported by Sharma et al. for sub-lineage EU t011 SCC*mec*IV, including *bla*Z*, mec*A*, tet*M*, aac*(A)-*aph*(D), *dfr*K, and *str*A (Sharma et al., 2016). In addition, this sub-lineage was associated with MRSA strains from a broad range of host species, including horses, cattle and pigs. In contrast to our results, none of the ST-398 isolates investigated by Sharma et al. carried any human-associated virulence genes (Sharma et al., 2016), supporting the idea of ongoing adaptive changes within this host generalist lineage.

In this study, three different homologs of *scn* were identified within the IEC (*scn*), SaPIbov5 (*scn*bov), and Saeq1 (*scn*eq), and three MGEs of a completely different nature and composition. Interestingly, 17/18 MRSA-ST398 of equine origin harbored two different SCIN-encoding variants (Figure 1). Inactivation of the C3 convertase complex in the alternative pathway of complement activation is considered one of the most important immune evasion strategies in *S. aureus* (Ricklin et al., 2009). A recent study on the biological effects of the equine variant of SCIN encoded by *scn*eq revealed its ability to block activation of the equine complement system, hence interfering with phagocytosis (de Jong et al., 2018).

Strikingly, *eq*SCIN was identified as a SCIN variant that functions in a much broader range of hosts, including horses, humans, and pigs (de Jong et al., 2018). In addition, our data might indicate a greater importance for contemporary carriage of allelic variants encoding Staphylococcal complement inhibitor (SCIN) for successful *S. aureus* niche- and host-adaption than previously thought, since complement activation is pivotal for *S. aureus* killing (Jongerius et al., 2007).

All MRSA ST398 positive for SAPIbov5 harbor two allelic variants encoding the von Willebrand binding protein, a potent activator of the blood prothrombin (Thomer et al., 2013). The SAPIbov5-encoded *vwb* variant shows specific activity toward equine and ruminant plasma (Viana et al., 2010). The presence of two, different vWbp-encoding genes might indicate a broadening in this genetic background of its flexibility with respect to host range. However, the functional role of the chromosomally-encoded *vwb* reported here, as well as the interplay and regulation of both variants requires further investigation.

Further research is warranted to track the origin and distribution of MGEs promoting niche adaption within the successful and zoonotic ST398 lineage, including genes facilitating resistance toward antimicrobials and biocides but also virulence and biofilm formation abilities (Feßler et al., 2010; Fessler et al., 2017; Wendlandt et al., 2013).

### Genomic integration site of phiNM3-like phages in equine MRSA-ST398 and hemolysis

The precise *hlb* integration site (Figure 2) of all phiNM3-like phages (sizes: 40,699 or 40,703 bp) in our study (5′-GTATCCGAATTGG-3′) has been described with respect to the ability of ΦSa3-like phages to integrate into *hlb* of ST398, an event which has rarely been reported (Kraushaar et al., 2017). A further recent retrospective study on human derived *S. aureus* isolates sharing the clonal complex 398 revealed 12 different phage types ranging in size from 40,712 bp up to 44,003 bp and four novel genomic integration sites for ΦSa3 phages (van Alen et al., 2018).

Interestingly, a prior study reported that isolates sharing an ST398 background were commonly found to have a significantly higher *hla* expression compared to other lineages, especially hospital-associated MRSA (Tavares et al., 2014). Notably, a strong expression of α-hemolysin on sheep blood agar might mask the lack of β-hemolysin production as a result of *hlb* disruption by ΦSa3 phages, a fact that might lead to overlooking an IEC-positive ST398 isolate in routine diagnostics and research (Figure 3).

## Conclusion

In equine MRSA-ST398, the contemporary presence of MGEs carrying genes known to encode virulence factors which have been associated with a certain degree of host-specificity, such as SAPIbov5 and β-hemolysin converting phages possessing an IEC variant, most likely mirrors the capacity to survive the initial stages of an invasive infection in different host species.

Further functional analyses are clearly needed to reveal the contribution of *scn* homologs and other immune modulating factors to host adaption, since the potential of equine MRSA-ST398 to cause severe infectious disease is clearly present.

Consequently, the spread of multi-drug resistant equine MRSA in veterinary clinics must by contained to enhance biosecurity for both veterinary staff and equine patients. This particular aspect of the “One Health” idea requires more emphasis in the development of targeted infection control strategies.

## Author contributions

BW and AL-B conceived and designed the experiments. K-SK, A-KB, and HG collected the data and samples. CH and FM performed laboratory analysis. BW, TS, RM, AL-B, KT, and HG analyzed the data. BW, KT AL-B, and HG wrote the article. All authors have read and approved the final draft of the manuscript.

### Conflict of interest statement

The authors declare that the research was conducted in the absence of any commercial or financial relationships that could be construed as a potential conflict of interest.

## Supplementary material

Supplementary Table 1 Whole genome sequences: accession numbers. Single nucleotide polymorphism (SNP) ranges for 18 MRSA-ST398-t011-SCC*mec*IV of equine origin. As a reference (Ref.), WGS data (CP020019) from an MRSA-ST398 (08S00974) isolated from a pig was included.

Supplementary Table 2 Single nucleotide polymorphism-distance matrix.
