# Supplementary material for: Equine Methicillin-Resistant Sequence Type 398 Staphylococcus aureus (MRSA) Harbor Mobile Genetic Elements Promoting Host Adaptation
Source: Front Microbiol. 2018 Oct 24;9:2516. doi: 10.3389/fmicb.2018.02516 (PMC6207647; doi:10.3389/fmicb.2018.02516)
Supplement: Supplementary Table 1 — Whole genome sequences: accession numbers. Single nucleotide polymorphism (SNP) ranges for 18 MRSA-ST398-t011-SCCmecIV of equine origin. As a reference (Ref.), WGS data (CP020019) from an MRSA-ST398 (08S00974) isolated from a pig was included. [file Table_1.DOCX]

**Accessions of equine MRSA strains**

| SUBID | BioProject | BioSample | Accession | Organism |
| --- | --- | --- | --- | --- |
| SUB3720202 | PRJNA435710 | SAMN08584161 | PUTY00000000 | *Staphylococcus aureus* IMT33368 |
| SUB3720249 | PRJNA435710 | SAMN08584162 | PUTZ00000000 | *Staphylococcus aureus* IMT33391 |
| SUB3720251 | PRJNA435710 | SAMN08584169 | PUUA00000000 | *Staphylococcus aureus* IMT33826 |
| SUB3720252 | PRJNA435710 | SAMN08584184 | PUUB00000000 | *Staphylococcus aureus* IMT33828 |
| SUB3720255 | PRJNA435710 | SAMN08584198 | PUUC00000000 | *Staphylococcus aureus* IMT33861 |
| SUB3720257 | PRJNA435710 | SAMN08584209 | PUUD00000000 | *Staphylococcus aureus* IMT33862 |
| SUB3720270 | PRJNA435710 | SAMN08584211 | PUUE00000000 | *Staphylococcus aureus* IMT33997 |
| SUB3732327 | PRJNA435710 | SAMN08612623 | PUXH00000000 | *Staphylococcus aureus* IMT34080 |
| SUB3732332 | PRJNA435710 | SAMN08612624 | PUXI00000000 | *Staphylococcus aureus* IMT34209 |
| SUB3732336 | PRJNA435710 | SAMN08612625 | PUXJ00000000 | *Staphylococcus aureus* IMT34426 |
| SUB3732339 | PRJNA435710 | SAMN08612626 | PUXK00000000 | *Staphylococcus aureus* IMT36995 |
| SUB3732353 | PRJNA435710 | SAMN08612628 | PUXL00000000 | *Staphylococcus aureus* IMT37082 |
| SUB3732355 | PRJNA435710 | SAMN08612631 | PUXM00000000 | *Staphylococcus aureus* IMT37264 |
| SUB3732360 | PRJNA435710 | SAMN08612645 | PUXN00000000 | *Staphylococcus aureus* IMT37277 |
| SUB3732364 | PRJNA435710 | SAMN08612647 | PUXO00000000 | *Staphylococcus aureus* IMT37325 |
| SUB3732383 | PRJNA435710 | SAMN08612658 | PUXP00000000 | *Staphylococcus aureus* IMT37340 |
| SUB3732386 | PRJNA435710 | SAMN08612661 | PUXQ00000000 | *Staphylococcus aureus* IMT37510 |
| SUB3797642 | PRJNA435710 | SAMN08731663 | PYBG00000000 | *Staphylococcus aureus* IMT37426 |
